# Viral Infection in the Development and Progression of Pediatric Acute Respiratory Distress Syndrome

**DOI:** 10.3389/fped.2016.00128

**Published:** 2016-11-24

**Authors:** Steven Nye, Richard J. Whitley, Michele Kong

**Affiliations:** ^1^The University of Alabama at Birmingham, Birmingham, AL, USA

**Keywords:** RSV, influenza A virus, H1N1 subtype, ARDS, pediatrics, viral infections

## Abstract

Viral infections are an important cause of pediatric acute respiratory distress syndrome (ARDS). Numerous viruses, including respiratory syncytial virus (RSV) and influenza A (H1N1) virus, have been implicated in the progression of pneumonia to ARDS; yet the incidence of progression is unknown. Despite acute and chronic morbidity associated with respiratory viral infections, particularly in “at risk” populations, treatment options are limited. Thus, with few exceptions, care is symptomatic. In addition, mortality rates for viral-related ARDS have yet to be determined. This review outlines what is known about ARDS secondary to viral infections including the epidemiology, the pathophysiology, and diagnosis. In addition, emerging treatment options to prevent infection, and to decrease disease burden will be outlined. We focused on RSV and influenza A (H1N1) viral-induced ARDS, as these are the most common viruses leading to pediatric ARDS, and have specific prophylactic and definitive treatment options.

## Introduction

Acute respiratory distress syndrome (ARDS) was first described in 1967 in adults presenting with tachypnea, hypoxemia, and decreased pulmonary compliance (1). Since then, the understanding, diagnosis, and management of ARDS has advanced greatly, with most research performed in adults. For more than two decades, pediatric health-care providers have relied heavily on adult-derived diagnostic criteria (2, 3). In recent years, the Pediatric Acute Lung Injury Consensus Conference (PALICC) has provided age-specific diagnostic and management guidelines for pediatric ARDS (4, 5). With these new pediatric-specific recommendations, future research in pediatric ARDS looks promising, as new areas for investigation have been identified (5).

One area of interest warranting further exploration is the role of viral infection in the development and progression of pediatric ARDS. While some information is available specific to pediatrics, much of our understanding continues to be derivative from adult data. This review outlines what is known about ARDS attributable to viral infections, specifically respiratory syncytial virus (RSV) and influenza A (H1N1) virus, as well as viral-specific treatment options.

## Epidemiology of RSV and Influenza-Induced ARDS

Accurate incidence outcomes reporting for pediatric ARDS are limited by the changing definition for ARDS. Studies using the 1994 American European Consensus Conference (AECC) definition (2) outlined epidemiology and outcomes for both acute lung injury (ALI) and ARDS as separate entities (6). However, both the Berlin definition (3) and the PALICC recommendations (5) no longer identify ALI as a separate entity, instead categorizing ARDS as mild, moderate, or severe. Since these guidelines are recent, few studies have been conducted to determine the incidence of ARDS utilizing these new criteria (7, 8). In addition, management for ARDS has also changed over the years, with improved outcomes demonstrated with lung-protective mechanical ventilation strategies (9, 10).

Pediatric studies conducted using the AECC definition define an incidence ranging from 2.96 to 12.8 per 100,000 children for all causes of ALI/ARDS, with the predominant etiology reported to be secondary to pneumonia, with or without systemic infection (11). In a more recent study, using the Berlin definition, Barreira et al. reported that ARDS accounts for 10% of all PICU admissions, and was associated with a high mortality rate of 24.5% (7). While the overall incidence of respiratory virus infection, in particular RSV and influenza A (H1N1) virus, leading to lower respiratory tract disease is widely studied (12, 13), the frequency of progression to pediatric ARDS has yet to be clearly determined.

Respiratory syncytial virus infection has been recognized as an important cause of lower lung disease. In an earlier study, Dahlem et al. reported 15.9% of ARDS cases due to RSV-related infection, but specific mortality for this group was not reported (14). In a more recent study by Lopez-Fernandez et al., 19.8% of patients admitted to the PICU with ARDS tested positive for RSV, with a reported mortality of 13.7% (15). A 12-year study in the Netherlands by Schene et al. analyzed 155 patients mechanically ventilated for RSV with 129 (83%) patients progressing to ARDS (16). Of those with ARDS, 38% were found to have a bacterial coinfection. The mortality rate was not used as a measure of outcome and therefore not reported.

Since the beginning of the influenza A (H1N1) virus pandemic of 2009, influenza-related respiratory failure has become a notable cause of ARDS (17). During the pandemic and post-pandemic era, it is clear that children are particularly vulnerable to disease even if they had been previously healthy (18). In 2009 alone, more than 43,000 cases were reported, with an estimated 73% of cases occurring in patients less than 24 years of age (13). In previous years, influenza-related pediatric deaths averaged 82 annually but increased to 317 during the 2009 pandemic (19). While post-pandemic studies suggest a decrease in influenza A (H1N1) virus disease severity and burden (20, 21), it continues to be a significant cause of severe illness and pediatric ARDS (22).

In a retrospective analysis of adult patients within the German ARDS network, investigators reported that 32% of ARDS patients were influenza A (H1N1) virus positive (23). In another pediatric study in India, Kinikar et al. reported that 18% of patients hospitalized with confirmed influenza A (H1N1) virus developed ARDS (24). In this study, 9 of the 15 children who died were found to have histologic pulmonary findings reflective of ARDS at autopsy. In Argentina, Farias et al. studied 147 patients admitted with respiratory failure due to influenza A (H1N1) virus and found 118 (80%) met criteria for pediatric ARDS, 45% of whom died within 28 days after PICU admission (25).

The second, less common novel influenza virus, avian influenza A (H5N1) virus, was first identified in 1998 (26) and remains a common cause of severe respiratory disease (27). Kawachi et al. reviewed pediatric patients with ARDS over a 4½-year period in Vietnam and found 12 (32.4%) of the 37 patients to have confirmed infection with the highly pathogenic influenza A (H5N1) virus (28). They described rapid progression of disease to ARDS with nine (75%) resulting in death. Further investigation has led to improved understanding of transmission, predominantly direct avian-to-human transmission with significant risk in handling sick or dead poultry. Type 2 pneumocytes and macrophages are the primary lung target (29).

Together, these studies demonstrate that RSV and influenza virus infection play a role in the development of pediatric ARDS. However, to better understand the disease burden, future studies should seek to more clearly identify the rate of occurrence of primary viral-induced ARDS, as well as incidence of secondary viral-induced lung injury. Furthermore, in patients who have a coinfection with a bacterial pathogen, it may be hard to determine whether the virus or bacteria played the inciting role in the development and progression of pediatric ARDS.

Taken together, the overall mortality attributable to either RSV or influenza is relatively similar; thus, it is more likely the syndrome of ARDS and associated pathology that is responsible for outcome.

## Other Viruses Leading to ARDS

While RSV and influenza A (H1N1) virus are the most commonly reported viruses leading to pediatric ARDS, other viral pathogens are worth mentioning. Typically viral infections leading to respiratory failure in the ICU are separated as community acquired and nosocomial (30).

Community acquired viral infections include both seasonal and pandemic pathogens (31). Seasonal viruses most commonly include RSV, non-pandemic influenza, rhinoviruses, parainfluenza, adenovirus, coronaviruses, and human metapneumovirus (hMPV). Seasonal viruses remain the most frequent cause of childhood community acquired pneumonia (32). The most common etiology of pediatric ARDS is primary pneumonia, with or without systemic infection (15). It can then be assumed that viral infections may play an important role in development of pediatric ARDS. However, determining an accurate estimate of the disease burden of viral-induced pediatric ARDS will be difficult, as many simple viral infections can progress to coinfection with the second virus or a bacterial pathogen. As will be detailed below, with the development of multiplex PCR diagnostic platforms that identify multiple viral agents, further insight into coinfections will develop.

A single-center adult study reported seven patients developing ARDS from adenovirus, four of whom died (33). Hung and Lin described a case of a 9-month-old male with adenoviral ARDS requiring extracorporeal membrane oxygenation (ECMO) (34). Hasvold et al. studied adult patients hospitalized with hMPV and discovered progression to ARDS in 19 (14.8%) patients (35). In 2014, an outbreak of enterovirus D68 in the United States led to symptoms of respiratory failure similar to influenza A (H1N1) virus, although less severe (36). While seasonal viruses typically cause severe infection in immunocompromised patients (37), cases of severe disease in immunocompetent patients are becoming increasingly reported (38).

At an international level, community acquired, novel pathogens have been recognized as a significant cause of ARDS in the last 15–20 years (31). In 2015, the World Health Organization (WHO) developed a panel of experts to prioritize emerging pathogens to likely cause severe outbreaks in the near future, and for which little or no preventative or curative treatments are available (39). The list includes two novel coronaviruses, severe acute respiratory syndrome (SARS)-CoV and MERS-CoV, which are widely recognized as noteworthy causes of ARDS.

In 2002, SARS-CoV led to the development of SARS in China (40). Affecting patients of all ages, SARS led to significant mortality worldwide within a few months (41). A large number of infected patients developed severe complications, with 20% developing ARDS (42). However, reported SARS cases have ceased since 2004 as the spread of infection has subsided (43). More recently, the second novel coronavirus, MERS-CoV, led to the Middle East respiratory syndrome (44). Clinical symptoms range from mild upper respiratory symptoms to severe pneumonia and ARDS, septic shock, and multi-organ failure (45) and carry an estimated mortality of 40% (46). This virus continues to be a substantial etiology of ARDS with high mortality as no definitive prevention or treatment other than supportive care has been identified (47).

With the unpredictable nature of epidemics and pandemics, these novel viruses illustrate the need to improve our understanding of viral progression to ARDS in order to advance management and reduce mortality.

Aside from community acquired viral infections, nosocomial infections are an important cause of respiratory illness, and can lead to ARDS in both adults and children. In mechanically ventilated adults, reactivation of latent herpes simplex virus (HSV) in the oropharynx can potentially lead to lower respiratory tract infection and ARDS (48, 49). However, the pathogenicity of reactivated HSV lower respiratory tract infection may not be that straightforward as it remains unclear whether HSV contributes to worsening illness or whether reactivation occurs due to the underlying critical illness (50). Schuller et al. found higher levels of clinical severity and mortality in critically ill immunocompetent adults with HSV-1 infection compared to immunocompromised patients with HSV-1 (51). The true extent of HSV reactivation in critically ill children leading to respiratory illness has yet to be studied. Hennus et al. described two previously healthy children presenting with respiratory failure due to human herpes virus 6 (HHV-6), and later workup revealed an immunodeficiency in both patients (52). A separate pediatric case reported a child with HSV ARDS resulting in need of extracorporeal support (53). These cases illustrate the rare, but possible severe infection and progression to ARDS from HSV-1.

Finally, many seasonal and pandemic viruses are a potential nosocomial infectious risk secondary to either a health-care provider or air-ventilation transmission. In a study over two influenza seasons in Germany, Huzly et al. reported a rate of nosocomial transmission of 24% (2012–2013) and 20% (2013–2014) (54). Specific guidelines are available to help prevent transmission of infectious pathogens through isolation precautions (55). However, Dhar et al. found that an increased number in patients placed on contact isolation led to a decrease in compliance with isolation precautions (56). Decreasing nosocomial transmission within care areas for critically ill patients is an important area for improvement.

## Diagnosis of Viral-Induced ARDS

The PALICC has recently provided guidelines for diagnosing pediatric ARDS (5). The new guidelines define important diagnostic criteria, including age, timing, origin of edema, imaging, and oxygenation. Patients with perinatal lung disease are excluded, and pARDS criteria must be met within 7 days of a clinical insult. The cause of respiratory failure must not be explained by heart failure or fluid overload, and must be evidenced by new pulmonary infiltrate(s) on chest radiograph consistent with parenchymal disease. Finally, the use of oxygenation index is preferred over the PaO_2_:FiO_2_ (PF) ratio in determining the severity of pARDS in mechanically ventilated patients, while the PF ratio or SpO_2_:FiO_2_ (SF) ratio may be used in patients requiring non-invasive ventilation (5). The same clinical guideline is used in the diagnosis of viral-induced pediatric ARDS. Currently, there are several different types of laboratory tests that are commercially available for diagnosis. Most clinical laboratories utilize antigen detection tests, which consist of multiple steps to accurately identify a single virus (57), with or without cell culture.

It is worth noting that over the past 20 years, the development and refinement of real-time reverse transcriptase polymerase chain reaction (RT-PCR) has enhanced the clinician’s ability to diagnose an array of viruses rapidly and accurately. Multiplex RT-PCR testing analyzes a single sample for multiple viral agents and subtypes simultaneously, producing sensitive and specific results in a short period of time (58). Even with the 2009 influenza A (H1N1) virus pandemic, the Centers for Disease Control and Prevention (CDC) quickly modified standard PCR assays to detect the new virus (59). A challenge to routine RT-PCR testing in all patients who present with viral symptoms is the prohibitive cost, need for specialized equipment, and the relatively longer time between sampling and availability of results (60). Furthermore, PCRs detect viral genes that are used as a surrogate measurement of whole virions. In some instances, viral gene detection may actually reflect non-replicating, non-infectious virions. Newer rapid point-of-care PCRs are currently being developed, but their implications for clinical decision making remain uncertain (61). In addition, rapid antigen detection tests (RADT) are also available commercially for detection of both RSV and influenza virus infection in the outpatient and emergency department settings (62, 63). However, in a recent study by Moesker et al., RADTs were found to have relatively low sensitivity compared to RT-PCR testing which limits their use for clinical decision making (64). Nonetheless, RADT maybe a valuable tool, especially during an outbreak, because it is a point-of-care test that is easy to use with a rapid turnaround time (65). Since clinical symptoms for different viral respiratory infections are often the same, and with the limitations of our current testing methods, it is critical that clinicians obtain microbiology data early, especially in the risk population (66–69).

There is also a large variability of disease severity in children infected with RSV or influenza A (H1N1) virus. In RSV infection, development of lower respiratory track disease in premature infants, with or without chronic neonatal lung disease is associated with a significantly higher risk of hospitalization, intensive care unit admission, need for mechanical ventilation, and death (12, 70–73). In a study of 2,147 children with lower respiratory infection due to RSV, Rodriguez et al. reported age less than 6 months, history of prematurity, chronic respiratory disease or congenital heart disease, and coinfection with adenovirus were significant predictors of increased disease severity (74). Similar predictors exist for children infected with influenza A (H1N1) virus, including age less than 5 years, a history of chronic lung disease, congenital heart disease, and immune compromise (Table 1) (75). It is therefore prudent that clinicians should conduct laboratory evaluations early in the illness for viral infections in these at-risk populations presenting with respiratory failure and ARDS.

**Table 1:** **Clinical risk factors for severe viral respiratory infection**.

RSV	Influenza A (H1N1) virus
Age <6 months	Age <5 years
History of prematurity	Chronic lung disease
Chronic lung disease	Congenital heart disease
Congenital heart disease	Immunocompromised conditions
Viral coinfection	

In contrast to clinical predictors of disease severity, the contribution of viral factors to disease burden remains unclear. In RSV infection, although earlier studies suggested no correlation between viral load and disease severity (76, 77) newer findings suggest otherwise. Studies by both DeVincenzo et al. and Houben et al. reported a direct correlation between viral load and disease severity in infants with primary RSV infection (78, 79). El Saleeby et al. also reported that viral load is independently associated with increased risk of patients with RSV requiring prolonged hospitalization or intensive care, or to develop respiratory failure (80). The relevance of viral load in influenza A (H1N1) virus infection is unclear. Launes et al. found that in children who had more than 5 days of symptoms, a higher influenza A (H1N1) viral load at diagnosis correlated with an increased risk of requiring mechanical ventilation (81). Similarly others have found that patients with systemic symptoms and pneumonia had higher viral load when compared to those with uncomplicated upper respiratory tract infections alone (82). As would be expected children have a higher influenza A (H1N1) viral load compared to adults because of less exposure to influenza antigens. However, this finding did not correlate with the occurrence of disease complications (83).

## Pathophysiology and Histology of RSV and Influenza A (H1N1) Viral-Induced ARDS

Both RSV and influenza A (H1N1) virus result in a broad spectrum of disease, ranging from mild upper respiratory symptoms to fulminant respiratory failure and ARDS (59, 84). This high degree of variability may be due to the pathogenicity of the viral pathogen, host immune response, or a combination of both (85).

Human RSV consists of subgroups A and B and primarily infects humans. The RSV genome encodes 11 different proteins involved in transmission, infection, evasion of host response, and replication (86). Infection is typically restricted to respiratory epithelial cells, including both type I and type II alveolar pneumocytes, from the trachea to the level of bronchioles. Infection leads to epithelial and interstitial inflammation with progression to inflammatory infiltrates and epithelial sloughing (87). After infection and viral replication, RSV causes epithelial cells to fuse, forming a syncytium from which the virus spreads from cell to cell (88). Those infected epithelial cells are then destroyed, releasing inflammatory cytokines and chemokines that ultimately attract additional inflammatory cells and degrade capillary integrity (89). Disruption of the alveolar–capillary barrier results in leakage of plasma proteins into interstitial tissue and within the alveoli, finally interfering with surfactant function (90). With an understanding of the pathophysiologic process of RSV, it is no surprise that progression to ARDS is a potential end point.

Aside from viral pathogenicity, host immune-mediated factors also contribute to disease severity. A rapidly progressive area of research is in understanding the role of biomarkers not only in the diagnosis and prognosis of ARDS, but also in potential therapeutic options to alter such biomarkers (91). Inflammatory proteins in the matrix metalloproteinase (MMP) family have been shown to be elevated in pediatric ALI (92) with a specific increase in MMP-9 production in RSV infection. Blocking MMP-9 *in vitro* and *in vivo* resulted in decrease in viral load (93). In addition, activation of several chemokine and interleukin subtypes, as well as tumor necrosis factors, has been shown to positively correlate with severity of illness in children with RSV (94). In their study, Fernandez et al. discovered that higher levels of soluble interleukin-10 (IL-10) positively correlated with both disease severity and duration of supplemental oxygen in infants with acute RSV infection (95). A separate study confirmed this associate with increased levels of IL-10 in nasopharyngeal secretions (96), but the pathogenicity of this correlation has yet to be determined.

Antigenic variability exists with both influenza A (H1N1) virus and the resultant immune-mediated response. Like influenza B and C, influenza A is made up of structural proteins and two groups of surface glycoproteins, hemagglutinin (HA), and neuraminidase (NA). These glycoproteins are responsible for attachment and entry into cells, viral spread throughout the respiratory tract, and are capable of a large degree of variability (97). Waterfowl serve as the largest natural reservoir for influenza A subtypes (98). The avian-to-human leap can occur through direct transmission (99) or, alternatively, through pigs (100). Although transmission from pigs to humans is a rare event, it occurred in the 1918 pandemic (101) a small outbreak in New Jersey in 1976 (102) and the most recent pandemic of influenza A (H1N1) starting in 2009 (103).

Influenza A (H1N1) virus primarily targets alveolar epithelial cells that serve as first-line defense against respiratory infections (104). Histological evaluation of 100 fatal cases of influenza A (H1N1) virus infection revealed diffuse alveolar damage with inflammation, fibrosis and edema, disruption of surfactant production, and detection of viral antigens throughout the lung parenchyma (105). Like RSV, this pathogenic can process rapidly progresses to refractory hypoxemia and ARDS (106).

In addition to viral pathogenicity, disease severity of influenza A (H1N1) virus infection is also closely associated with host response (107). In both healthy patients and those with comorbidities, influenza A (H1N1) virus can lead to an exaggerated inflammatory response with dysregulation of local and systemic chemokine and cytokine production (108, 109). In a study specifically in the pediatric population, Takano et al. found a positive correlation with disease severity and elevated levels of serum interferon gamma, several interleukin types, and monocyte chemoattractant protein-1 (MCP-1) (110). Some studies suggest that these elevations may be adequate immune response to infection (111), but further exploration of the inflammatory response in influenza A (H1N1) virus infection is likely to yield development of potential interventions to prevent disease progression to ARDS.

## Treatment Options Available for RSV and Influenza A (H1N1) Viral-Induced ARDS

The treatment of ARDS has significantly evolved over the past several decades. Perhaps the greatest improvement in management developed from studies involving lung-protective ventilation (9). Other interventions such as inhaled nitric oxide (112), fluid management (113), use of steroids (114), and prone positioning (115) are being further investigated and validated. However, specific treatment for RSV infection remains lacking. Despite the substantial short and long-term morbidity and mortality associated with RSV disease in children, the current management for RSV infection consists of supportive care, in the form of oxygen supplementation, adequate hydration, and mechanical ventilation for those who develop respiratory failure. Multiple therapeutic strategies have been explored with very limited success, and a vital need remains for an effective disease treatment.

Interventions for either virus can be separated into primary prevention of infection, typically through vaccination, and reduction of infectious burden once transmission has already taken place.

Primary prevention through vaccination continues to be a major area of research and potential advancement for both RSV and influenza A (H1N1) virus. To date, no RSV vaccine has proven efficacious. While significant research has been devoted to vaccine development, major obstacles specific to RSV, such as young age of infection, lack of persistent immunity, and poorly validated animal models make it difficult to find an effective and safe solution (116). One current prospect, Medi-534, a live-attenuated, intranasal vaccine providing protection against both RSV and parainfluenza 3, although safe in children ages 1–9, has yet to show a beneficial immunogenic response in infants (117). Other more recent advances continue to focus on live-attenuated vaccines, as well as chimeric live vectors (118), with varying antibody response between children (119, 120).

While vaccine development continues, prophylactic use of polyclonal RSV intravenous immunoglobulin (RespiGam) or human anti-F monoclonal antibodies (palivizumab and motavizumab – which is not yet licensed for use) in high-risk infants has been shown to reduce the risk of RSV-associated acute lower respiratory tract infections and disease severity (121). Palivizumab is a human, monoclonal antibody targeted to block viral infected cells from fusing with adjacent cells (122). Palivizumab has been shown to be most effective in high-risk populations, specifically premature infants and those with chronic lung disease or congenital heart disease (123). The use of palivizumab as treatment for RSV infection in mechanically ventilated pediatric patients has not been shown to be effective (124). Furthermore, studies have also shown that palivizumab prophylaxis in these patients has a limited effect on the total disease burden of RSV infection, including overall RSV-related hospital admissions and resource utilization (12, 125). Although not approved for use in the United States, motavizumab, the second-generation derivative of palivizumab, decreased viral load compared with placebo (126). However, in a more recent study of hospitalized RSV infected infants treated with motavizumab or placebo, no antiviral effect was demonstrated (127). Furthermore, both therapies produce only temporary, passive immunity (128).

The 2009 influenza A (H1N1) virus was a novel strain, leaving children and young adults with little if any preexisting antibodies and without adequate protection with the seasonal influenza vaccine alone (129). A new influenza A (H1N1) virus vaccine was rapidly developed and has subsequently been shown to be safe and effective at providing adequate immunological response (130, 131). One post-pandemic study showed a correlation with higher rates of influenza A (H1N1) virus infection, compared with other influenza types, along with increased ICU admissions for countries with limited numbers of the population having received influenza A (H1N1) virus vaccination (132).

In addition to general supportive care, the second goal of therapy in viral infection focuses on reducing the infectious burden and, theoretically, subsequent viral sequelae. Currently, inhaled ribavirin is the only approved antiviral treatment for RSV infection in children (133) but its use is associated with potential teratogenicity, and its efficacy remains uncertain (134). Ribavirin directly and indirectly inhibits replication of both DNA and RNA viruses, including RSV (135). Studies in infants found a decrease in mortality and respiratory deterioration, and a decrease in days of hospitalization and days of mechanical ventilation in ventilated infants (136). Luo et al. reported an adult case of severe RSV infection progressing to ARDS that was successfully treated with inhaled ribavirin (137), but overall effectiveness in treatment of viral pediatric ARDS has yet to be determined. In addition to its use in RSV, ribavirin has also been used in treatment of severe influenza A (H1N1) virus infection (138). Ribavirin can be given orally but is typically aerosolized when used for respiratory viral infections. However, safety considerations regarding potential teratogenicity and exposure to health-care workers during administration (134) limit its use. The American Academy of Pediatrics does not recommend the routine use of ribavirin to treat RSV infection, reserving its use for patients with potentially life-threatening disease (139). Several small molecule inhibitors that interfere with RSV F protein (MDT-637 and JNJ-2408068) (140) have been identified, including the GS-5806 that was recently evaluated in a challenge safety study of healthy adults (141). In this study by DeVincenzo et al., treatment resulted in decreased viral burden and severity of clinical disease. The use of these small molecule inhibitors in the context of pediatric subjects who develop ARDS remains untested at this point.

Neuraminidase inhibitors (which prevent the release of influenza virions), including oral oseltamivir, inhaled zanamivir and laninamivir, and parenteral peramivir, remain first-line interventions for influenza. Only oseltamivir, peramivir, and zanamivir are available in the United States. Perhaps the most widely used, oseltamivir results in a significant decrease in duration of symptoms as well as severity of illness with early treatment (<48 h of symptoms) (142). Use of oseltamivir in severe cases of influenza A (H1N1) has become standard practice (24, 143), although some studies have shown increased resistance (144). Randolph et al. conducted a retrospective study of 838 children admitted to the PICU with confirmed influenza A (H1N1) infection (145). Overall, 564 (67.3%) required mechanical ventilation, but the rate of progression to ARDS was not reported. Although 88% were treated with oseltamivir, there was no association with improved mortality. Farias et al. found a reduced mortality in patients with PARDS from influenza A (H1N1) if oseltamivir was administered within the first 24 h (25).

While further studies are needed to look at the effectiveness of antiviral medications in the treatment of viral-induced ARDS, in recent years, investigators are also focusing on the potential benefits of immune modulation. With enhancement in research surrounding viral pathogenicity and host immune response, potential targets of intervention will hopefully be identified.

Aside from viral-specific therapies, ECMO has been utilized as rescue therapy for severe respiratory failure in pediatrics for more than 20 years, with more than 50% survival (146). The overall use of ECMO for treatment of ARDS has increased with improvement in mortality (147). The recent PALICC recommendations conclude that ECMO should be considered for treatment of pARDS when lung-protective strategies have failed, when the cause of respiratory failure is thought to be reversible, or when the child may be suitable for lung transplantation (148).

The use of ECMO in pediatric respiratory failure due to RSV is well reported (149, 150). In their retrospective review of 151 children requiring mechanical ventilation for RSV bronchiolitis, Flamant et al. reported the use of ECMO in 14 patients (151). In this study, the median duration of ECMO was 12.5 (5–18) days with a survival rate of 71.4%. On the other hand, the use of ECMO in pARDS due to influenza A (H1N1) virus is sparsely reported (152, 153) and most of our understanding stems from adult studies. In a study in Australia and New Zealand during the 2009 influenza A (H1N1) pandemic, 68 adult patients with influenza-induced ARDS were treated with ECMO (154). The median duration of ECMO was 10 (7–15) days. When the report was submitted, 48 (71%) patients had survived to ICU discharge, with 14 deaths and 6 patients remaining in the ICU, 2 of whom remained on ECMO. In their study in the United Kingdom during the same pandemic, Noah et al. discovered a decrease in mortality for patients with ARDS due to influenza A (H1N1) who were referred and transferred to an ECMO center compared with matched non-ECMO-referred patients (155). In this study, 69 patients received ECMO with a mortality rate of 14.4%. Expanded use of ECMO within the pediatric population for influenza A (H1N1) virus induced ARDS has yet to be investigated. However, the use of ECMO in refractory cases or RSV or influenza A (H1N1) virus induced ARDS should be considered when applicable.

## Conclusion

While it is clear that viral infections are an important cause of pediatric ARDS, the exact disease burden remains unknown. With more definitive diagnostic criteria, clinicians now have a wide array of research possibilities regarding pediatric ARDS, both retrospective and prospective. Further studies to expand our understanding of viral-induced pediatric ARDS will be of great benefit, both in understanding the epidemiology and viral-specific treatment options available. In addition, an improved comprehension of viral transmission, pathogenicity, and host response will be particularly important in times of pandemics, either from known or novel viruses. Finally, continued efforts in prevention and treatment of viral infections will likely be of greatest advantage to decrease viral progression to pediatric ARDS.

## Author Contributions

SN, RW, and MK contributed to the conception, writing, and final edits of this manuscript.

## Conflict of Interest Statement

The authors declare that the research was conducted in the absence of any commercial or financial relationships that could be construed as a potential conflict of interest.
